# Efficacy and safety of *Cordyceps sinensis* (*Hirsutella sinensis*, Cs-C-Q80) in chronic bronchitis

**DOI:** 10.3389/fphar.2024.1428216

**Published:** 2024-08-13

**Authors:** Xinyang Shu, Dongfeng Xu, Yumin Qu, Xiaofeng Shang, Kehong Qiao, Cuiling Feng, Hongsheng Cui, Xianping Zhao, Yuxin Li, Yu Peng, Demin Li, Hongchun Zhang

**Affiliations:** ^1^ National Center for Respiratory Medicine, State Key Laboratory of Respiratory Health and Multimorbidity, National Clinical Research Center for Respiratory Diseases, Institute of Respiratory Medicine, Chinese Academy of Medical Sciences, Department 1 of Traditional Chinese Medicine Pulmonary Disease, Center of Respiratory Medicine, China-Japan Friendship Hospital, Beijing, China; ^2^ Department of Respiratory and Critical Care Medicine, The First Affiliated Hospital of Nanyang Medical College, Nanyang, China; ^3^ Department of Respiratory, Beijing Xuanwu Hospital of Traditional Chinese Medicine, Beijing, China; ^4^ Department of Respiratory and Critical Care Medicine, Taiyuan Central Hospital, Taiyuan, China; ^5^ Department of Traditional Chinese Medicine, Peking University People’s Hospital, Beijing, China; ^6^ Department of Respiratory, The Third Affiliated Hospital of Beijing University of Chinese Medicine, Beijing, China; ^7^ Department of Respiratory, Changzhi People’s Hospital, Changzhi, China; ^8^ Department of Respiratory, Beijing Huairou Hospital of Traditional Chinese Medicine, Beijing, China; ^9^ Department of Rehabilitation, People’s Hospital of Qitaihe City, Qitaihe, China

**Keywords:** Cordyceps sinensis, bailing capsule, chronic bronchitis, randomized controlled trial, traditional Chinese medicine

## Abstract

**Background:** *Cordyceps sinensis* is a traditional Chinese medicine that has shown promise for the management of chronic bronchitis (CB). We aim to assess the efficacy and safety of a preparation of *C sinensis* named Bailing capsule (*Hirsutella sinensis*, Cs-C-Q80) compared with a placebo in patients with CB.

**Methods:** This randomized, double-blind, placebo-controlled, parallel-group clinical trial (Chinese Clinical Trial Registry; registration number: ChiCTR1900025707) recruited patients with CB from eight hospitals in China between May 2019 and December 2020. Patients were randomized 2:1 to receive Bailing capsule or a placebo orally for 48 weeks (2.0 g, three times a day).

**Results:** Among 240 patients who were randomized, 238 (Bailing capsule: 159, placebo: 79) were included in the primary analysis. Bailing capsule significantly reduced the frequency of acute exacerbation of CB (AECB) compared with the placebo during treatment (0.43 ± 0.82 vs. 1.56 ± 1.34; *P* < 0.001) and follow-up (0.21 ± 0.64 vs. 0.45 ± 0.93; *P* = 0.026). Bailing capsule improved the severity of expectoration (*P* = 0.046) and wheezing (*P* = 0.010) in AECB during follow-up. The severity of CB after treatment was significantly improved in the Bailing capsule group compared with the placebo group (*P* = 0.035), particularly in terms of expectoration (*P* = 0.012) and wheezing (*P* = 0.003). The risk of adverse events, mainly including infectious and invasive diseases and gastrointestinal symptoms, did not significantly differ between the two groups (29.6% vs. 30.4%).

**Conclusion:** In patients with CB, Bailing capsule significantly reduces the frequency of AECB and ameliorates the severity of AECB and CB symptoms.

**Clinical Trail Registration:** https://www.chictr.org.cn, identifer ChiCTR1900025707.

## Introduction

Chronic bronchitis (CB) is characterized by persistent cough and sputum production for a minimum of 3 months per year, consecutively for 2 years, without any other underlying conditions accounting for these symptoms. It is caused by chronic inflammation of the respiratory tract, leading to excessive mucus production and secretion, and impaired mucociliary clearance (Valipour et al., 2020; Bigot et al., 2022; Krimsky et al., 2023). Its main clinical manifestations are persistent cough, excessive sputum production, and/or shortness of breath. In population-based studies, the prevalence of CB varies from 3.6% to 16.7% (Maas et al., 2020; Puddu et al., 2021; Frederiksen et al., 2022; Jarhyan et al., 2022). Studies have indicated a significant correlation between CB and deteriorating health status (Kim et al., 2011), and it is significantly associated with a faster decline in forced expiratory volume in 1 s (FEV_1_) and increased mortality rates (Ekberg-Aronsson et al., 2005; Guerra et al., 2009). While various medical management options have been developed for CB, there is insufficient evidence to recommend the routine use of medications, including antibiotics, bronchodilators, and mucolytic agents, for effective symptom relief in CB (Malesker et al., 2020). Therefore, patients still face high risks for acute exacerbation of the disease (Hartman et al., 2021).

Bailing capsule contains fermented *C. sinensis* mycelium powder (*Hirsutella sinensis*, Cs-C-Q80), which mainly comprises adenosine, mannitol, ergosterol, polysaccharides, a variety of amino acids, vitamins, and trace elements, and thus can be used as a substitute for wild *C. sinensis*. It tonifies the lungs and kidneys and nourishes the essence and qi. It is used for cough, asthma, hemoptysis, and lumbago caused by lung and kidney deficiencies. In addition, it is used as an adjuvant treatment for CB and chronic renal insufficiency (Paterson, 2008). *Cordyceps sinensis* improves airway inflammation in patients with CB by modulating the levels of inflammatory factors, thereby alleviating clinical symptoms (Hsu et al., 2008). Immunodeficiency is the fundamental cause of acute exacerbation of CB (AECB) (Xu and Li, 2010). Bailing capsule improves immune function by supplementing essential amino acids and cordycepic acid. Therefore, Bailing capsules may improve the prognosis of CB by exerting multiple effects, including anti-inflammatory and anti-hypoxic effects, inhibition of airway remodeling, and enhancement of immune function (Xu and Li, 2010). Based on these theoretical foundations, we hypothesized that Bailing capsule may improve the prognosis of CB.

## Methods

### Study design and oversight

This randomized, double-blind, placebo-controlled, multi-center clinical trial (XXX) was performed in eight clinical centers in China. According to a stratified randomization method, subjects were allocated into a Bailing capsule group and a placebo group in a 2:1 ratio. The study adhered to the principles of Good Clinical Practice and the Declaration of Helsinki, and ethics approval was obtained from the ethics committee of China-Japan Friendship Hospital (no: 2018–168-K122). All patients or their legal representatives signed informed consent before randomization.

### Study population

Patients were included if they met: 1) patients who met the diagnostic criteria for CB; 2) age between 18 and 75 years old (including 18 and 75), no gender restrictions; 3) the main symptom scores of CB were ≤1 point, including cough, expectoration, and wheezing; and 4) having experienced at least two AECBs within the past year (confirmed by medical records). CB was diagnosed according to the guidelines for the diagnosis and treatment of cough published by the Chinese Medical Association Respiratory Branch in 2015. The details diagnostic criteria for CB include: 1) persistent cough and sputum production for more than 2 years, with a cumulative or continuous duration of at least 3 months each year, with no other causes of chronic cough; 2) if the duration of symptoms is less than 3 months per year, but there is clear objective evidence (e.g., from X-rays or pulmonary function tests), a diagnosis can still be made.

The exclusion criteria were as follows: 1) exacerbation of CB within the past month; 2) coexisting diseases such as pulmonary tuberculosis, eosinophilic bronchitis, bronchogenic carcinoma, idiopathic pulmonary fibrosis, bronchial asthma, bronchiectasis, congestive heart failure, chronic pharyngitis, posterior nasal drip syndrome, or gastroesophageal reflux disease; 3) patient’s FEV_1_ as a percentage of the predicted value is less than 80% after inhalation of bronchodilators; 4) coexisting severe primary disease, including cardiovascular, cerebrovascular, or hematopoietic disorder; 5) serum alanine aminotransferase (ALT), aspartate aminotransferase (AST), or serum creatinine (Scr) levels are higher than 1.5 times the upper limit of the normal reference range; and 6) pregnant or lactating women.

### Randomization and blinding

According to the randomization, patients from each center were assigned to either the Bailing capsule or placebo group in a 2:1 ratio. The randomization numbers were computer-generated. For each randomization number, the corresponding packaging number of the study drug was automatically blinded. Prior to drug dispensing, the researchers applied the corresponding study drug using the randomization number through the system. All participants involved in the trial, including patients, doctors, nurses, on-site investigators, and members of the clinical endpoint committee, were unaware of the treatment assignment.

### Procedures

The patients in the Bailing capsule group were randomly assigned to receive Bailing capsules [batch number: 1,811,114 (Expiry date: October 2021); 1,902,127 (Expiry date: January 2022); 1,906,001 (Expiry date: May 2022) four capsules, 2.0 g, orally] three times a day for 48 weeks. The patients were followed up for an additional 48 weeks up after treatment. The placebo group received matching placebo capsules [batch number: 1,812,002 (Expiry date: January 2022)] specifically developed for the Bailing capsule trial. The placebo capsules had the same appearance as the Bailing capsules and a similar taste and odor when opened. When AECB occurs, conventional treatment may involve the use of antibiotics, cough suppressants, and expectorants. Traditional Chinese herbal decoctions or patent medicines can also be used for the treatment of AECB, but each course of treatment should not exceed 21 days, and the total duration of treatment should not exceed 3 months. The baseline patient characteristics, including clinical presentations, vital signs, and laboratory test results, were collected from case report forms.

### Outcomes

The primary outcome was the frequency of AECB, which was defined as: 1) at least two episodes of cough, expectoration, and wheezing that worsened; and 2) a minimum interval of 1 week between the two exacerbations. The secondary outcomes included the time of first AECB occurrence, interval between AECB occurrences, duration of AECB, severity of AECB (cough, expectoration, and wheezing), CB symptom score (cough, expectoration, and wheezing), pulmonary function, imaging examination, immunological indicators, and adverse events.

Severity of AECB: Cough, expectoration, and wheezing were classified into normal, mild, moderate, and severe based on severity scores of 0, 1, 2, and 3, respectively. Details of the criteria for AECB severity are summarized in Supplementary Table S1.

CB symptom score: During the treatment period, the subjects were evaluated weekly for cough, sputum, and wheezing using a visual analog scoring method. Subjects marked a corresponding scale on a 10-cm line to indicate the severity of cough, expectoration, and wheezing based on their own perception. A score of 0 indicated no related symptoms, whereas a score of 10 indicated extremely severe symptoms that were intolerable. The duration of symptoms (cough, expectoration, and wheezing) with a score ≥4 for CB was also assessed (Martin Nguyen et al., 2021).

Pulmonary function: Pulmonary function tests include tests of FEV_1_, forced vital capacity (FVC), FEV_1_ as a percentage of the predicted value (FEV_1_%pred), and the ratio of FEV_1_ to FVC (FEV_1_/FVC). Other measurements include maximum mid-expiratory flow (MMEF) and peak expiratory flow (PEF).

Imaging examination: Computed tomography (CT) scans were used to evaluate small airway lesions in the lungs, the presence of emphysema, and other complications.

Immunological indicators: To determine the total CD3^+^ T cell count, CD3^+^CD4^+^ T cell subset, CD3^+^CD8^+^ T cell subset, and CD4+/CD8+ T cell ratio, changes in immune function indicators were assessed based on the normal reference range used in each center’s laboratory for each parameter.

Adverse events: Adverse events during treatments were assessed through physical examination, laboratory tests, liver and kidney function test, and electrocardiography.

### Statistical analysis

In a previous study, the effective rate of Bailing capsule in treating CB was 85%, whereas the control group had an effective rate of 65% (Liu et al., 2006). Considering a dropout rate of 20%, the minimum sample size required for each group was 75 cases (80% power, two-sided α = 0.05 and β = 0.20). The characteristics of patients in the Bailing capsule and placebo groups included continuous and categorical data. Continuous data are presented as mean (standard deviation) or median (range) according to the data distribution, whereas categorical data are shown as frequency and proportion. Groups were compared using an independent samples *t*-test or Wilcoxon rank-sum test for continuous data, whereas categorical data were compared using the chi-square test or Fisher’s exact test. All statistical tests were performed using a two-tailed test, and significance was set at *P* ≤ 0.05. All statistical analysis were performed using SPSS 26.0 (SPSS, Chicago, IL, United States).

## Results

**FIGURE 1:**
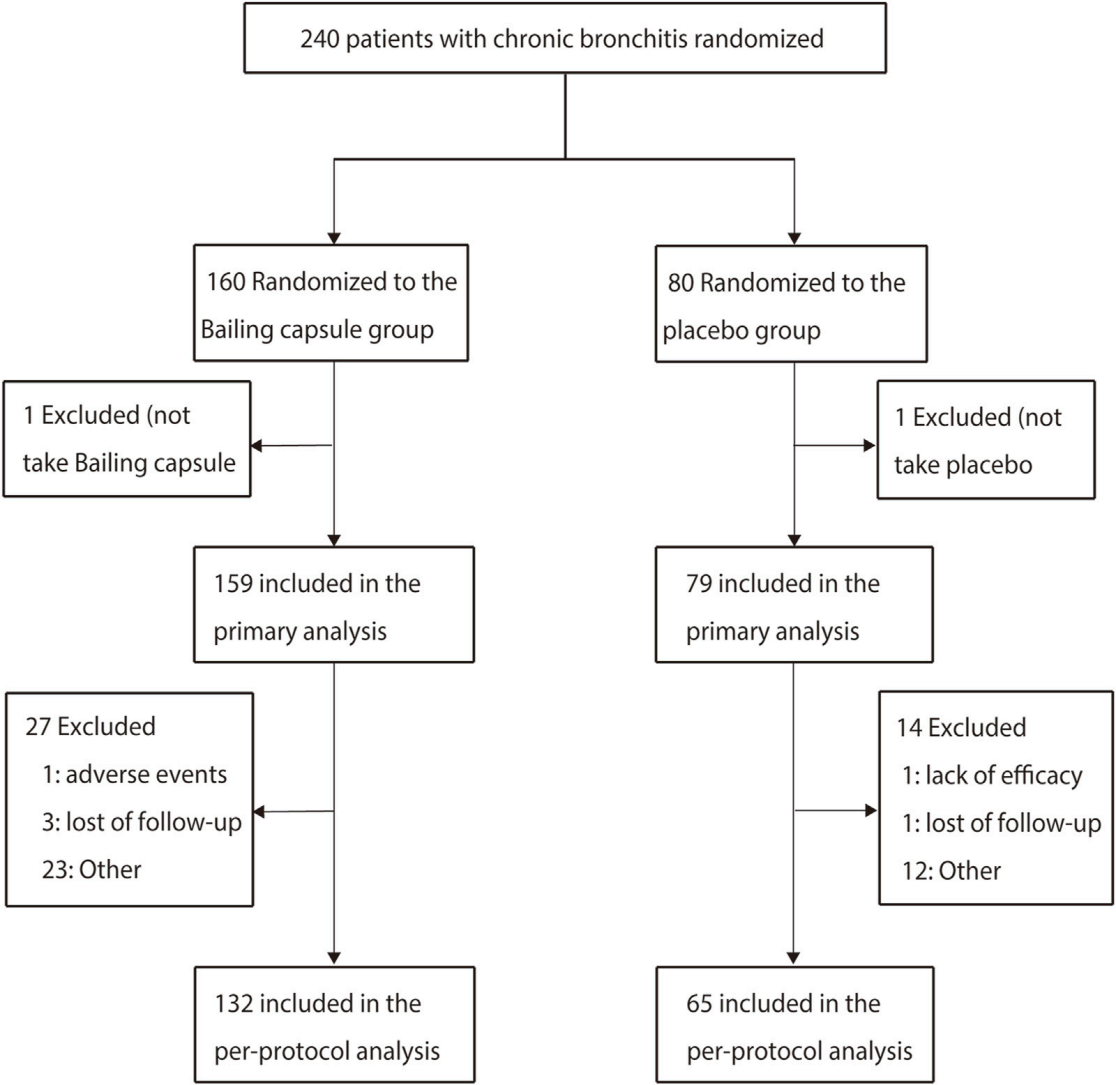
Patient screening process used in this study.

Between May 2019 and December 2020, a total of 240 patients were enrolled from eight hospitals in China, among whom 160 patients assigned to the Bailing capsule group and 80 to the placebo group. Two patients did not take the study drugs (one in the Bailing capsule group and one in the placebo group), leaving 238 patients (159 in the Bailing capsule group and 79 in the placebo group) for the primary analysis. A total of 197 patients (41 patients were excluded, including 27 in the Bailing capsule group and 14 in the placebo group) were included in the confirmatory per-protocol analysis (Figure 1).

### Baseline characteristics

Most baseline patient characteristics, including age, sex, ethnicity, height, number of AECBs in the past year, number of hospitalizations due to AECBs in the past year, medical history, allergy history, family history, body temperature, pulse rate, respiratory rate, and diastolic blood pressure, were balanced between the Bailing capsule and placebo groups (Table 1). The mean age of the patients included was 57.6 years, and 49.2% were male. The frequency of using concomitant medications for respiratory diseases during the trial period is shown in Supplementary Table S2.

**TABLE 1:** Baseline characteristics of enrolled patients.

Variable	Bailing capsule (N = 159)	Placebo (N = 79)	*P*-value
Age, mean (SD), years	58.06 ± 10.51	56.65 ± 11.92	0.352
Sex			0.155
Male (%)	73 (45.91)	44 (55.70)	
Female (%)	86 (54.09)	35 (44.30)	
Ethnicity			1.000
Han (%)	154 (96.86)	76 (96.20)	
Other (%)	5 (3.14)	3 (3.80)	
Height (cm)	164.44 ± 7.79	165.15 ± 8.83	0.526
Weight (kg)	67.94 ± 10.79	71.23 ± 10.79	0.028
Number of AECB in the past year	2.52 ± 0.61	2.52 ± 0.64	0.970
Number of hospitalizations due to AECB in the past year	0.11 ± 0.31	0.08 ± 0.31	0.469
Medical history (%)			0.615
Yes	86 (54.09)	40 (50.63)	
No	73 (45.91)	39 (49.37)	
Allergy history (%)			0.302
Yes	19 (11.95)	6 (7.59)	
No	140 (88.05)	73 (92.41)	
Family history (%)			0.445
Yes	15 (9.43)	10 (12.66)	
No	144 (90.57)	69 (87.34)	
Body temperature (°C)	36.39 ± 0.28	36.34 ± 0.32	0.211
Pulse rate (beats/min)	72.09 ± 8.92	70.00 ± 9.66	0.100
Respiratory rate (breaths/min)	18.60 ± 2.11	18.46 ± 2.10	0.626
SBP (mmHg)	127.75 ± 9.38	131.08 ± 12.00	0.020
DBP (mmHg)	80.74 ± 6.20	81.24 ± 7.27	0.582

AECB: acute exacerbation of chronic bronchitis; DBP: diastolic blood pressure; SBP: systolic blood pressure; SD:standard deviation.

### Primary outcome

After 48 weeks, the mean number of AECBs in the Bailing capsule group (0.43 ± 0.82) was significantly lower than that in the placebo group (1.56 ± 1.34; *P* < 0.001). Similarly, during the follow-up period, the mean number of AECBs in the Bailing capsule group (0.21 ± 0.64 was significantly lower than that in the placebo group (0.45 ± 0.93; *P* = 0.026) (Table 2).

**TABLE 2:** Primary and Secondary outcomes.

Outcome	Bailing capsule (N = 159)	Placebo (N = 79)	*P*-value
Primary outcome			
Number of AECB during treatment	0.43 ± 0.82	1.56 ± 1.34	<0.001
Number of AECB during follow-up	0.21 ± 0.64	0.45 ± 0.93	0.026
Secondary outcomes			
Time of first occurrence of AECB (days)	135.94 ± 173.74	92.88 ± 105.28	0.685
Interval between AECB occurrence (days)	151.97 ± 122.28	153.22 ± 113.23	0.858
Duration of AECB (days)	36.51 ± 70.47	26.23 ± 42.01	0.971
Severity of AECB during treatment			
Cough	2.21 ± 0.46	1.98 ± 0.27	<0.001
Expectoration	2.00 ± 0.51	2.02 ± 0.13	0.925
Wheezing	1.25 ± 1.04	1.37 ± 0.73	0.567
Severity of AECB during follow-up			
Cough	2.28 ± 0.46	2.00 ± 0.50	0.053
Expectoration	1.76 ± 0.60	2.00 ± 0.00	0.046
Wheezing	0.96 ± 0.79	1.56 ± 0.77	0.010
Severity of CB after treatment	1.51 ± 1.98	2.55 ± 3.23	0.035
Cough	0.76 ± 0.90	1.13 ± 1.43	0.179
Expectoration	0.48 ± 0.75	0.86 ± 1.16	0.012
Wheezing	0.26 ± 0.67	0.56 ± 1.01	0.003
Severity of CB after follow-up	1.32 ± 2.48	1.16 ± 1.86	0.833
Cough	0.61 ± 0.98	0.49 ± 0.80	0.602
Expectoration	0.46 ± 0.96	0.49 ± 0.73	0.425
Wheezing	0.25 ± 0.92	0.19 ± 0.52	0.825
Pulmonary function			
FEV_1_ (mL)			
Baseline	2585.58 ± 608.99	2672.76 ± 723.35	0.585
After 48 weeks	2507.52 ± 623.64	2664.36 ± 776.60	0.161
After 96 weeks	2496.30 ± 786.54	2552.64 ± 654.56	0.462
Changes of FEV_1_ from after 48 weeks to baseline	−63.64 ± 292.16	−7.27 ± 351.67	0.527
Changes of FEV_1_ from after 96 weeks to baseline	−74.53 ± 534.23	−123.77 ± 360.63	0.419
FVC (mL)			
Baseline	3327.58 ± 773.24	3481.05 ± 1018.84	0.532
After 48 weeks	3280.97 ± 749.53	3478.73 ± 1063.12	0.219
After 96 weeks	3236.82 ± 807.98	3418.11 ± 999.22	0.220
Changes of FVC from after 48 weeks to baseline	−41.13 ± 335.71	−17.27 ± 351.60	0.912
Changes of FVC from after 96 weeks to baseline	−70.80 ± 328.99	−92.45 ± 335.04	0.688
MMEF (mL/s)			
Baseline	2642.93 ± 1160.19	2686.32 ± 1327.48	0.877
After 48 weeks	2456.11 ± 1151.26	2463.20 ± 1500.87	0.668
After 96 weeks	2621.68 ± 1387.06	2421.70 ± 1070.70	0.483
Changes of MMEF from after 48 weeks to baseline	−182.91 ± 1035.61	−242.44 ± 825.62	0.855
Changes of MMEF from after 96 weeks to baseline	22.19 ± 1317.07	−293.40 ± 1477.49	0.673
PEF (mL/s)			
Baseline	6300.65 ± 1823.56	6022.40 ± 1887.02	0.546
After 48 weeks	6086.44 ± 2093.94	5959.89 ± 1944.00	0.780
After 96 weeks	6181.84 ± 1787.54	6200.12 ± 1773.30	0.952
Changes of PEF from after 48 weeks to baseline	−143.97 ± 1790.84	75.16 ± 1675.82	0.886
Changes of PEF from after 96 weeks to baseline	−70.36 ± 1226.17	236.08 ± 2393.58	0.905
FEV_1_%pred			
Baseline	99.42 ± 14.37	96.87 ± 14.50	0.393
After 48 weeks	97.95 ± 18.58	97.43 ± 18.68	0.578
After 96 weeks	88.66 ± 18.02	89.84 ± 15.07	0.759
Changes of FEV_1_%pred from after 48 weeks to baseline	−1.77 ± 15.43	0.90 ± 13.73	0.559
Changes of FEV_1_%pred from after 96 weeks to baseline	−11.28 ± 18.48	−7.08 ± 15.80	0.222
FEV_1_/FVC (%)			
Baseline	79.08 ± 7.50	79.00 ± 7.39	0.417
After 48 weeks	76.30 ± 8.19	77.28 ± 6.53	0.781
After 96 weeks	76.74 ± 10.19	75.99 ± 8.74	0.758
Changes of FEV_1_/FVC from after 48 weeks to baseline	−2.71 ± 7.46	−1.39 ± 7.29	0.493
Changes of FEV_1_/FVC from after 96 weeks to baseline	−2.57 ± 10.40	−2.64 ± 9.94	0.863
Immunological indicators			
CD3^+^ T cells			
Baseline	67.58 ± 9.33	69.32 ± 8.90	0.118
After 48 weeks	66.58 ± 9.91	69.80 ± 8.22	0.044
Changes of CD3^+^ T cells from after 48 weeks to baseline	−0.60 ± 4.90	1.01 ± 6.91	0.152
CD3^+^CD4^+^ T cell subset			
Baseline	38.60 ± 8.33	39.39 ± 7.98	0.502
After 48 weeks	37.84 ± 7.79	38.92 ± 7.71	0.404
Changes of CD3^+^CD4^+^ T cell subset from after 48 weeks to baseline	−0.77 ± 5.37	−0.33 ± 4.63	0.415
CD3^+^CD8^+^ T cell subset			
Baseline	25.01 ± 8.79	25.81 ± 10.41	0.905
After 48 weeks	25.01 ± 8.51	26.49 ± 10.25	0.493
Changes of CD3^+^CD8^+^ T cell subset from after 48 weeks to baseline	0.27 ± 4.50	0.84 ± 2.89	0.333
CD4+/CD8+ T cell subset			
Baseline	1.81 ± 1.02	1.86 ± 1.03	0.656
After 48 weeks	1.55 ± 0.75	1.73 ± 0.99	0.582
Changes of CD4+/CD8+ T cell subset from after 48 weeks to baseline	−0.26 ± 0.80	−0.14 ± 0.52	0.588

### Secondary outcomes

*Time of first AECB occurrence*: The time of first AECB occurrence in the Bailing capsule group (135.94 ± 173.74 days) did not significantly differ from that in the placebo group (92.88 ± 105.28 days; *P* = 0.685) (Table 2).

*Interval between AECB occurrences:* The interval between AECB occurrences in the Bailing capsule group (151.97 ± 122.28 days) did not significantly differ from that in the placebo group (153.22 ± 113.23 days; *P* = 0.858) (Table 2).

*Duration of AECB*: AECB duration in the Bailing capsule group (36.51 ± 70.47 days) did not significantly differ from that in the placebo group (26.23 ± 42.01 days; *P* = 0.971) (Table 2).

*Severity of AECB*: During treatment, cough severity differed significantly between the Bailing capsule and placebo groups (*P* < 0.001), whereas the severity of expectoration (*P* = 0.925) and wheezing (*P* = 0.567) did not. During follow-up, cough severity no longer differed significantly between the Bailing capsule and placebo groups (*P* = 0.053), and Bailing capsule was associated with greater improvement in expectoration (*P* = 0.046) and wheezing (*P* = 0.010) than the placebo (Table 2).

*Severity of CB*: The overall severity of CB after treatment in the Bailing capsule group was significantly improved when compared with that in the placebo group (*P* = 0.035), particularly in terms of expectoration (*P* = 0.012) and wheezing (*P* = 0.003) (Table 2; Supplementary Figure S1). During follow-up, CB severity did not significantly differ between the Bailing capsule and placebo groups (*P* = 0.833), including cough (*P* = 0.602), expectoration (*P* = 0.425), and wheezing (*P* = 0.825) (Supplementary Figure S2). Bailing capsule significantly reduced the durations of cough, expectoration, and wheezing with scores ≥4 as compared with the placebo (Table 2).

*Pulmonary function*: The changes in FEV_1_, FVC, MMEF, PEF, FEV_1_%pred, and FEV_1_/FVC after treatment and during follow-up were not significantly different between the Bailing capsule and placebo groups (*P* > 0.050) (Table 2).

*Pulmonary emphysema*: At baseline, the proportions of participants with pulmonary emphysema in the Bailing capsule and placebo groups were 14.94% and 12.00%, respectively. After 48 weeks of treatment, these proportions were 13.91% and 13.56%, respectively, and after 96 weeks of treatment, they were 15.18% and 16.36%, respectively. There was no significant difference in the incidence of pulmonary emphysema between the Bailing capsule and placebo groups (*P* > 0.050).

*Immunological indicators*: The changes in CD3^+^ T cell count (*P* = 0.152), CD3^+^CD4^+^ T cell subset (*P* = 0.415), CD3^+^CD8^+^ T cell subset (*P* = 0.333), and CD4+/CD8+ T ratio (*P* = 0.588) did not significantly differ between the Bailing capsule and placebo groups (Table 2).

### Adverse events

The numbers of adverse events in Bailing capsule and placebo groups were 103 (47 patients) and 61 (24 patients) during treatments, and the numbers of serious adverse events were 6 (5 patients) and 6 (5 patients), respectively. There was no significant difference between the groups in terms of the risk of adverse events (*P* = 0.896) and serious adverse events (*P* = 0.258). Infectious and invasive diseases occurred in 29 cases, with a total of 45 occurrences, resulting in an incidence rate of 12.18% (20 cases with 28 occurrences in the Bailing capsule group, resulting in an incidence rate of 12.58%, and 9 cases with 17 occurrences in the placebo group, resulting in an incidence rate of 11.39%, *P* = 0.792). There were 21 cases of gastrointestinal diseases, with a total of 33 occurrences, resulting in an incidence rate of 8.82% (17 cases and 29 occurrences in the Bailing capsule group, with an incidence rate of 10.69%; 4 cases and 4 occurrences in the placebo group, with an incidence rate of 5.06%, *P* = 0.159).

## Discussion

This randomized, double-blind, placebo-controlled, multi-center clinical trial aimed to assess the efficacy and safety of Bailing capsule for CB. A total of 240 patients were recruited, and the patient characteristics were well balanced between the groups. The study revealed that Bailing capsule significantly reduced the number of AECBs compared to a placebo during treatment and follow-up. Moreover, the severity of AECBs during follow-up was significantly improved in the Bailing capsule group compared to the placebo group, particularly in terms of expectoration and wheezing. Furthermore, CB severity after treatment was significantly improved in the Bailing capsule group compared to the placebo group, particularly in terms of expectoration and wheezing. Similarly, the durations of cough, expectoration, and wheezing with symptom scores ≥4 were shorter in the Bailing capsule group than in the placebo group. There were no significant differences in pulmonary function and immunological indicators between the Bailing capsule and placebo groups. Finally, no significant differences in adverse events, which mainly included infectious and invasive diseases and gastrointestinal symptoms, were observed between the two groups.

Our study revealed that Bailing capsule reduced the number of AECBs as well as AECB and CB severity. AECB and CB severity mainly improved in terms of expectoration and wheezing. There are several reasons for the beneficial effects of Bailing capsule: 1) both animal experiments and clinical studies have demonstrated that Bailing capsule stabilizes the levels of inflammatory factors in the body of patients with chronic obstructive pulmonary disease (COPD) (Hsu et al., 2008). 2) The main components of Bailing capsule can correct disorders in amino acid, protein, and lipid metabolism, thereby promoting protein synthesis and improving cellular immune function. Its immunosuppressant action mainly involves inhibiting the function of antigen-presenting cells, blocking the delivery of external signal stimulation, achieving a state of low immune response, and affecting the ability of dendritic cells to stimulate proliferation to achieve immunosuppressive effects (Ma et al., 2007). 3) Bailing capsule can clear hydroxyl free radicals in the body, inhibit lipid peroxidation reactions, reduce aortic cholesterol deposition, and exert a protective effect on cells. Its main mechanism may involve activating monocytes, macrophages, T and B lymphocytes, and natural killer cells, reducing lipid peroxidation, and improving the activity of superoxide dismutase and glutathione peroxidase, thereby reducing tissue damage caused by free radicals and lipid peroxidation (Wang et al., 2012; Singh et al., 2013; Xiao et al., 2017). 4) Bailing capsule can inhibit the expression of fibrotic factors such as α-SMA, TGF-β, Snai, and CTGF and promote the modification of fibronectin, laminin, and COX-2 on the glomerular basement membrane. It can also inhibit the production of collagen and extracellular matrix, thereby exerting an anti-fibrotic effect (Xu and Li, 2010; Yang et al., 2018). Interestingly, we observed a significant decrease in the number of AECB in both groups after discontinuing therapy. This reduction could be attributed to the effective control measures implemented against COVID-19, which played a pivotal role in mitigating the occurrence of acute exacerbations in chronic respiratory conditions. Specifically, there is evidence indicating a fall in the frequency of acute exacerbations among COPD patients during the COVID-19 epidemic in China (Xu and Li, 2010; Yang et al., 2018).

Our study did not reveal any significant differences between Bailing capsule and the placebo in terms of the time of first occurrence of AECB, interval between AECB occurrences, AECB duration, and pulmonary function. The precise mechanism by which the specific active monomers of Bailing capsules exert their effects has yet to be confirmed. A large cohort study involving 5,002 participants showed that among 447 individuals with a history of chronic cough/phlegm at baseline, only 5% (23 cases) developed airflow limitation after a follow-up period of 5.8–11.4 years (median, 8.9 years), despite their risk being twice that of asymptomatic individuals (de Marco et al., 2007). In a cohort study involving 1,412 participants, a survival curve of patients with CB aged ≥50 years who developed airflow limitation showed that within a 5-year follow-up period, the majority of patients did not experience airflow limitation, whereas after 5 years, the survival curve sharply declined (Guerra et al., 2009). Therefore, lung capacity measurements cannot sensitively predict symptoms, exercise capacity, and overall quality of life, nor can they reflect disease heterogeneity. Only after irreversible pathological changes occur in the lungs, lung capacity crosses the threshold of disease (Agusti et al., 2010; Reyfman et al., 2018; Stolz et al., 2022). Interesting, we noted Bailing capsule was associated with high cough score in severity of AECB during treatment, which could explained by Bailing capsules show limited efficacy in reducing inflammation during acute phases. Consequently, relying solely on Bailing capsules may not effectively alleviate cough symptoms during acute exacerbation periods, and combination with other anti-inflammatory medications may be necessary to achieve optimal therapeutic outcomes (Li, 2019). Finally, Bailing capsules possesses the function of replenishing lung and kidney, as well as enhancing vital energy. It can regulate white blood cell levels in the body and exhibits a certain degree of anti-inflammatory effect. An elevated cough score during AECB treatment AECB might be attributed to Bailing capsules’ facilitation of sputum expectoration. Initially, it may increase coughing; however, this is actually a process of clearing the respiratory tract and alleviating inflammation, which could potentially be beneficial for long-term disease control (Zhang, 2019).

Immunological indicators did not significantly change upon using Bailing capsule. In previous studies, patients with CB who were prone to recurrent exacerbations showed no significant differences in cellular and humoral immune parameters when compared with an asymptomatic non-smoking control group, and CB patients showed no significant differences in lymphocyte subsets, serum immunoglobulins, and complement (C3 and C4) levels when compared with an adult control group (Fietta et al., 1988; Qvarfordt et al., 1998). CD8^+^ T cells are more abundant in the lungs of patients with COPD than in the general population, and their levels are negatively correlated with lung function. A decreased peripheral blood CD4/CD8 ratio may be associated with the occurrence of acute exacerbation of COPD (Kemeny et al., 1999; Hong and Xiao, 2023). Peripheral blood lymphocyte subsets showed no significant differences between patients with CB and the normal population, whereas CD8^+^ T cells and the CD4/CD8 ratio differed in COPD patients compared to the normal population. The use of Bailing capsule was not associated with any adverse events.

Several shortcomings of this study should be acknowledged. First, the background therapies for patients with CB are diverse, which may have affected the therapeutic effects of Bailing capsule. Second, the sample size was small, and stratified analyses according to patients’ characteristics were not performed, which requires further large-scale randomized controlled trials. Third, Bailing capsule is a traditional Chinese medicine, and the exact mechanism of action remains unclear. Finally, the effects of Bailing capsule on clinical outcomes related to the progression of CB were not investigated.

## Conclusion

The patented Chinese medicine Bailing capsule significantly reduces the number of AECB events and ameliorates the symptoms of AECB and CB in patients with CB. The therapeutic mechanism of Bailing capsule in CB requires further exploration.

## Data Availability

The original contributions presented in the study are included in the article/Supplementary Material, further inquiries can be directed to the corresponding author.

## References

[B1] Agusti A. Calverley P. M. A. Celli B. Coxson H. O. Edwards L. D. Lomas D. A. (2010). Characterisation of COPD heterogeneity in the ECLIPSE cohort. Respir. Res. 11, 122. 10.1186/1465-9921-11-122 20831787 PMC2944278

[B2] Bigot P. Chesseron S. Saidi A. Sizaret D. Parent C. Petit-Courty A. (2022). Cleavage of occludin by cigarette smoke-elicited cathepsin S increases permeability of lung epithelial cells. Antioxidants (Basel) 12, 5. 10.3390/antiox12010005 36670867 PMC9854811

[B3] de Marco R. Accordini S. Cerveri I. Corsico A. Antó J. M. Künzli N. (2007). Incidence of chronic obstructive pulmonary disease in a cohort of young adults according to the presence of chronic cough and phlegm. Am. J. Respir. Crit. Care Med. 175, 32–39. 10.1164/rccm.200603-381oc 17008642

[B4] Ekberg-Aronsson M. Pehrsson K. Nilsson J.-A. Nilsson P. M. Löfdahl C.-G. (2005). Mortality in GOLD stages of COPD and its dependence on symptoms of chronic bronchitis. Respir. Res. 6, 98. 10.1186/1465-9921-6-98 16120227 PMC1224873

[B5] Fietta A. Bersani C. De Rose V. Grassi F. A. Mangiarotti P. Uccelli M. (1988). Evaluation of systemic host defense mechanisms in chronic bronchitis. Respiration 53, 37–43. 10.1159/000195394 3260392

[B6] Frederiksen A. L. Laustsen B. H. Bælum J. Pedersen M. L. Bønløkke J. H. (2022). Prevalence of chronic obstructive pulmonary disease and chronic bronchitis among predominantly smoking workers in the seafood industry in Greenland. Int. J. Chron. Obstruct Pulmon Dis. 17, 1167–1177. 10.2147/COPD.S349106 35620348 PMC9128641

[B7] Guerra S. Sherrill D. L. Venker C. Ceccato C. M. Halonen M. Martinez F. D. (2009). Chronic bronchitis before age 50 years predicts incident airflow limitation and mortality risk. Thorax 64, 894–900. 10.1136/thx.2008.110619 19581277 PMC4706745

[B8] Hartman J. E. Garner J. L. Shah P. L. Slebos D.-J. (2021). New bronchoscopic treatment modalities for patients with chronic bronchitis. Eur. Respir. Rev. 30, 200281. 10.1183/16000617.0281-2020 33472961 PMC9488715

[B9] Hong X. Xiao Z. (2023). Changes in peripheral blood TBNK lymphocyte subsets and their association with acute exacerbation of chronic obstructive pulmonary disease. J. Int. Med. Res. 51, 3000605231182556. 10.1177/03000605231182556 37382080 PMC10328016

[B10] Hsu C.-H. Sun H.-L. Sheu J.-N. Ku M.-S. Hu C.-M. Chan Y. (2008). Effects of the immunomodulatory agent Cordyceps militaris on airway inflammation in a mouse asthma model. Pediatr. Neonatol. 49, 171–178. 10.1016/s1875-9572(09)60004-8 19133568

[B11] Jarhyan P. Hutchinson A. Khaw D. Prabhakaran D. Mohan S. (2022). Prevalence of chronic obstructive pulmonary disease and chronic bronchitis in eight countries: a systematic review and meta-analysis. Bull. World Health Organ 100, 216–230. 10.2471/BLT.21.286870 35261410 PMC8886252

[B12] Kemeny D. M. Vyas B. Vukmanovic-Stejic M. Thomas M. J. Noble A. Loh L. C. (1999). CD8(+) T cell subsets and chronic obstructive pulmonary disease. Am. J. Respir. Crit. Care Med. 160, S33–S37. 10.1164/ajrccm.160.supplement_1.10 10556167

[B13] Kim V. Han M. K. Vance G. B. Make B. J. Newell J. D. Hokanson J. E. (2011). The chronic bronchitic phenotype of COPD: an analysis of the COPDGene Study. Chest 140, 626–633. 10.1378/chest.10-2948 21474571 PMC3168856

[B14] Krimsky W. Neal Ii R. E. Kim V. (2023). Airway mucosal remodeling: mechanism of action and preclinical data of pulsed electric fields for chronic bronchitis and mucus hypersecretion. Respiration 102, 948–960. 10.1159/000534370 37906995

[B15] Li L. (2019). An exploration of the clinical efficacy of ambroxol hydrochloride injection in combination with kidney-tonifying, spleen-strengthening, lung-clearing, and asthma-relieving decoction in treating acute exacerbations of chronic bronchitis in elderly patients. Shanxi Med. J. 48, 1345–1346.

[B16] Liu K. Wang S. Geng Z. (2006). Observation on the therapeutic efficacy of Bailin capsules as an adjunct treatment for chronic bronchitis. Shandong Med. 32, 64. 10.3969/j.issn.1002-266X.2006.32.050

[B17] Ma L.-l. Yang X.-y. Gao J.-z. (2007). Experimental study on effect of Bailing Cpsule on dendritic cells in mice. Zhongguo Zhong Xi Yi Jie He Za Zhi 27, 905–908. 17990458

[B18] Maas A. Kothe H. Centeno I. P. Gutiérrez Leiva M. J. Dalhoff K. (2020). Prevalence of chronic bronchitis and respiratory health profile of a population exposed to wood smoke in Nicaragua. J. Health Pollut. 10, 200607. 10.5696/2156-9614-10.26.200607 32509408 PMC7269325

[B19] Malesker M. A. Callahan-Lyon P. Madison J. M. Ireland B. Irwin R. S. CHEST Expert Cough Panel (2020). Chronic cough due to stable chronic bronchitis: CHEST expert panel report. Chest 158, 705–718. 10.1016/j.chest.2020.02.015 32105719

[B20] Martin Nguyen A. Bacci E. D. Vernon M. Birring S. S. Rosa C. L. Muccino D. (2021). Validation of a visual analog scale for assessing cough severity in patients with chronic cough. Ther. Adv. Respir. Dis. 15, 17534666211049743. 10.1177/17534666211049743 34697975 PMC8552382

[B21] Paterson R. R. M. (2008). Cordyceps: a traditional Chinese medicine and another fungal therapeutic biofactory? Phytochemistry 69, 1469–1495. 10.1016/j.phytochem.2008.01.027 18343466 PMC7111646

[B22] Puddu P. E. Menotti A. Kromhout D. Kafatos A. Tolonen H. (2021). Chronic bronchitis in the 50-year follow-up of the European cohorts of the Seven Countries Study: prevalence, mortality and association with cardiovascular diseases. Respir. Med. 181, 106385. 10.1016/j.rmed.2021.106385 33848923

[B23] Qvarfordt I. Riise G. C. Larsson S. Almqvist G. Rollof J. Bengtsson T. (1998). Immunological findings in blood and bronchoalveolar lavage fluid in chronic bronchitis patients with recurrent infectious exacerbations. Eur. Respir. J. 11, 46–54. 10.1183/09031936.98.11010046 9543269

[B24] Reyfman P. A. Washko G. R. Dransfield M. T. Spira A. Han M. K. Kalhan R. (2018). Defining impaired respiratory health. A paradigm shift for pulmonary medicine. Am. J. Respir. Crit. Care Med. 198, 440–446. 10.1164/rccm.201801-0120pp 29624449 PMC6118019

[B25] Singh M. Tulsawani R. Koganti P. Chauhan A. Manickam M. Misra K. (2013). Cordyceps sinensis increases hypoxia tolerance by inducing heme oxygenase-1 and metallothionein via Nrf2 activation in human lung epithelial cells. Biomed. Res. Int. 2013, 569206. 10.1155/2013/569206 24063008 PMC3770031

[B26] Stolz D. Mkorombindo T. Schumann D. M. Agusti A. Ash S. Y. Bafadhel M. (2022). Towards the elimination of chronic obstructive pulmonary disease: a Lancet Commission. Lancet 400, 921–972. 10.1016/S0140-6736(22)01273-9 36075255 PMC11260396

[B27] Valipour A. Fernandez-Bussy S. Ing A. J. Steinfort D. P. Snell G. I. Williamson J. P. (2020). Bronchial rheoplasty for treatment of chronic bronchitis. Twelve-month results from a multicenter clinical trial. Am. J. Respir. Crit. Care Med. 202, 681–689. 10.1164/rccm.201908-1546OC 32407638 PMC7462406

[B28] Wang J. Liu Y.-M. Cao W. Yao K.-W. Liu Z.-Q. Guo J.-Y. (2012). Anti-inflammation and antioxidant effect of Cordymin, a peptide purified from the medicinal mushroom Cordyceps sinensis, in middle cerebral artery occlusion-induced focal cerebral ischemia in rats. Metab. Brain Dis. 27, 159–165. 10.1007/s11011-012-9282-1 22327557

[B29] Xiao Y. Huang Q. Zheng Z. Guan H. Liu S. (2017). Construction of a Cordyceps sinensis exopolysaccharide-conjugated selenium nanoparticles and enhancement of their antioxidant activities. Int. J. Biol. Macromol. 99, 483–491. 10.1016/j.ijbiomac.2017.03.016 28274870

[B30] Xu H. Li S. (2010). Pharmacological effects of Bailing capsule and its application in lung disease research. Zhongguo Zhong Yao Za Zhi 35, 2777–2781. 10.4268/cjcmm20102030 21246839

[B31] Yang L. Jiao X. Wu J. Zhao J. Liu T. Xu J. (2018). Cordyceps sinensis inhibits airway remodeling in rats with chronic obstructive pulmonary disease. Exp. Ther. Med. 15, 2731–2738. 10.3892/etm.2018.5777 29456676 PMC5795554

[B32] Zhang D. (2019). Clinical study on Bailing Capsules combined with cefoperazone and sulbactam in treatment of acute exacerbation of chronic bronchitis. Drugs and Clin. 34, 98–101.

