# Supplementary material for: Efficacy and safety of Cordyceps sinensis (Hirsutella sinensis, Cs-C-Q80) in chronic bronchitis
Source: Front Pharmacol. 2024 Aug 13;15:1428216. doi: 10.3389/fphar.2024.1428216 (PMC11347402; doi:10.3389/fphar.2024.1428216)
Supplement: Supplementary file 1 [file DataSheet1.docx]

Supplementary Material

# Supplementary Tables

**Online Data Supplement**

Table S1. The criteria for the severity of AECB

| **Symptom** | **Normal (0 score)** | **Mild (1 score)** | **Moderate (2 score)** | **Severe (3 score)** |
| --- | --- | --- | --- | --- |
| Cough | no cough | only morning cough | cough throughout the day | frequent cough |
| Expectoration | no obvious phlegm | 10-50ml of phlegm throughout the day and night or clear and easily coughed up | 50-100ml of phlegm throughout the day and night or yellow and thick and easily expectorated | over 100ml of phlegm throughout the day and night or yellow and thick and difficult to expectorate |
| Wheezing | no obvious wheezing | occasional wheezing that does not affect sleep or activities | wheezing during daily activities but not during rest | wheezing that prevents lying flat and significantly affects sleep and activities |

Table S2. The frequency of concomitant medication use for respiratory diseases during the trial period

|  | Bailing capsule (N=159) | | |  | Placebo (N=79) | | |  | Total (N=238) | | |  |
| --- | --- | --- | --- | --- | --- | --- | --- | --- | --- | --- | --- | --- |
| Drugs | events | Case | (%) |  | events | Case | (%) |  | events | Case | (%) |  |
| Total | 72 | 33 | 20.75 |  | 51 | 16 | 20.25 |  | 123 | 49 | 20.59 |  |
| Adrenocortical hormone | 26 | 18 | 11.32 |  | 17 | 9 | 11.39 |  | 43 | 27 | 11.34 |  |
| Expectorants | 15 | 13 | 8.18 |  | 15 | 8 | 10.13 |  | 30 | 21 | 8.82 |  |
| Antitussive drug | 12 | 9 | 5.66 |  | 4 | 4 | 5.06 |  | 16 | 13 | 5.46 |  |
| Phosphodiesterase inhibitor | 6 | 6 | 3.77 |  | 1 | 1 | 1.27 |  | 7 | 7 | 2.94 |  |
| M-choline receptor antagonists | 4 | 3 | 1.89 |  | 4 | 3 | 3.80 |  | 8 | 6 | 2.52 |  |
| Antiasthmatic drugs-other | 4 | 4 | 2.52 |  | 2 | 2 | 2.53 |  | 6 | 6 | 2.52 |  |
| β-adrenoceptor agonists | 4 | 3 | 1.89 |  | 8 | 2 | 2.53 |  | 12 | 5 | 2.10 |  |
| Allergic medium blockers | 1 | 1 | 0.63 |  | 0 | 0 | 0.00 |  | 1 | 1 | 0.42 |  |


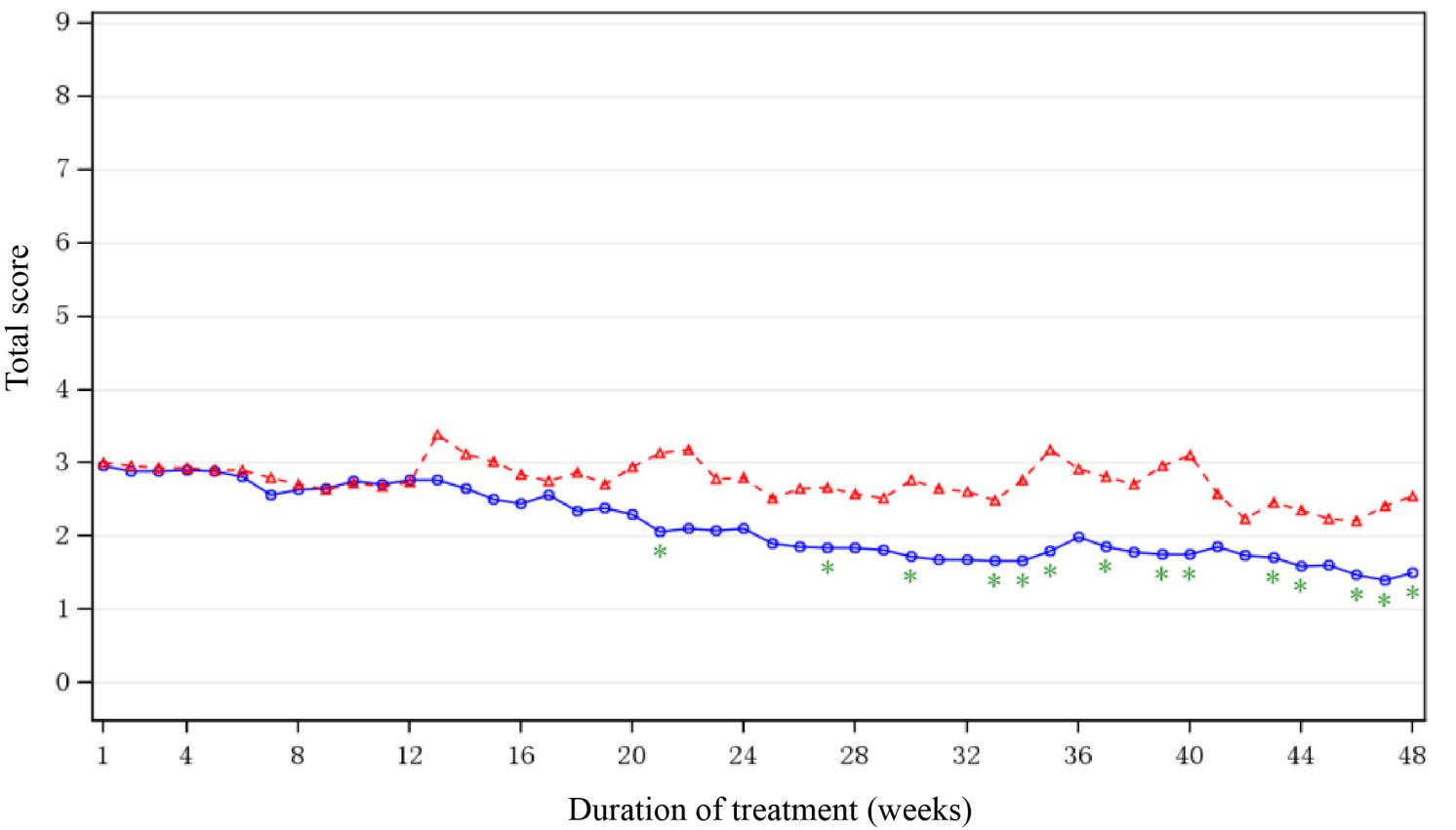


Figure S1. Changes in the total score of chronic bronchitis symptoms evaluation within 48 weeks of treatment


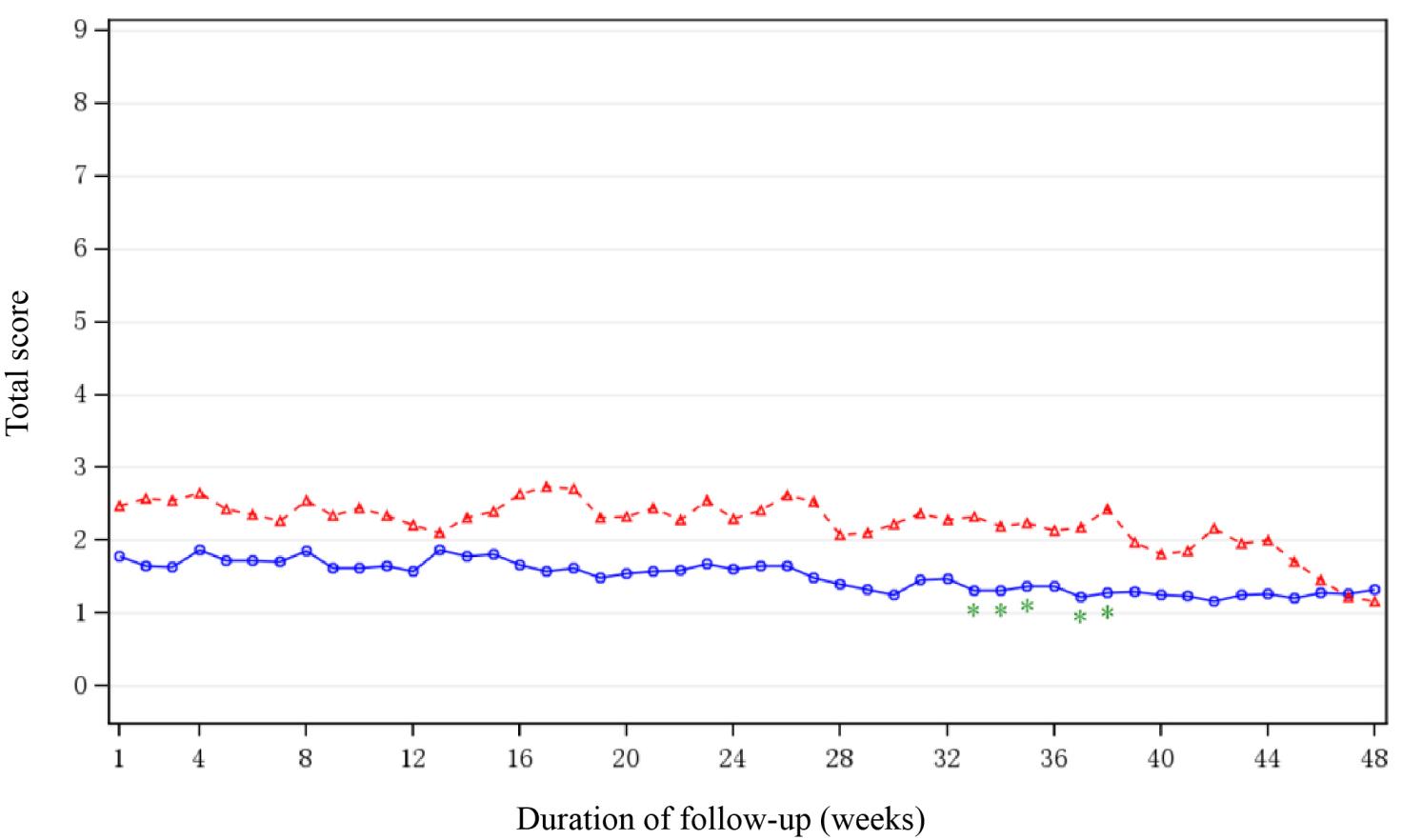


Figure S2. Changes in the total score of chronic bronchitis symptoms evaluation within 48 weeks of follow-up
